# CRISPR-mediated editing of β-lactoglobulin (*BLG*) gene in buffalo

**DOI:** 10.1038/s41598-024-65359-9

**Published:** 2024-06-27

**Authors:** Aseem Tara, Priyanka Singh, Devika Gautam, Gaurav Tripathi, Chirag Uppal, Shreya Malhotra, Sacchinandan De, Manoj K. Singh, Bhanu P. Telugu, Naresh L. Selokar

**Affiliations:** 1 Animal Biotechnology Division (ABTD), ICAR-National Dairy Research Institute, Karnal, Haryana 132001 India; 2 Division of Animal Science, University of Missouri, Columbia, MO 65211 USA

**Keywords:** Buffalo, CRISPR/Cas, Milk allergy, β-Lactoglobulin (BLG), Cloned embryos, Biological techniques, Biotechnology, Developmental biology, Molecular biology, Zoology

## Abstract

Milk is a good source of nutrition but is also a source of allergenic proteins such as α-lactalbumin, β-lactoglobulin (BLG), casein, and immunoglobulins. The Clustered Regularly Interspaced Short Palindromic Repeats (CRISPR)/Cas technology has the potential to edit any gene, including milk allergens. Previously, CRISPR/Cas has been successfully employed in dairy cows and goats, but buffaloes remain unexplored for any milk trait. In this study, we utilized the CRISPR/Cas9 system to edit the major milk allergen BLG gene in buffaloes. First, the editing efficiency of designed sgRNAs was tested in fibroblast cells using the T7E assay and Sanger sequencing. The most effective sgRNA was selected to generate clonal lines of *BLG*-edited cells. Analysis of 15 single-cell clones, through TA cloning and Sanger sequencing, revealed that 7 clones exhibited bi-allelic (−/−) heterozygous, bi-allelic (−/−) homozygous, and mono-allelic (−/+) disruptions in *BLG*. Bioinformatics prediction analysis confirmed that non-multiple-of-3 edited nucleotide cell clones have frame shifts and early truncation of BLG protein, while multiple-of-3 edited nucleotides resulted in slightly disoriented protein structures. Somatic cell nuclear transfer (SCNT) method was used to produce blastocyst-stage embryos that have similar developmental rates and quality with wild-type embryos. This study demonstrated the successful bi-allelic editing (−/−) of *BLG* in buffalo cells through CRISPR/Cas, followed by the production of *BLG*-edited blastocyst stage embryos using SCNT. With CRISPR and SCNT methods described herein, our long-term goal is to generate gene-edited buffaloes with BLG-free milk.

## Introduction

Domesticated buffaloes play a significant role in dairy industry. Globally, buffaloes contribute 16% of fresh whole milk, while cattle contribute to the remainder 84%. In Southeast Asia, the role of buffaloes is even more pronounced, accounting for 35.45% of fresh whole milk, while cattle contribute 64.54%^1^. Milk, a prominent nutritious food, holds significant importance in daily diets. However, some proteins such as α-lactalbumin, β-lactoglobulin (BLG), casein, and immunoglobulins act as allergens to susceptible individuals^2^. Among these proteins, BLG is a major allergen in the milk of farm animals, including buffaloes^3^. Traditional efforts to reduce milk allergenicity involve processing methods like pasteurization or ultra-high temperature treatment to denature milk proteins^2^. However, these methods are costly, impact nutritional value, and could potentially maintain allergenicity by creating new epitopes^4^. Alternatively, recently developed gene editing (GE) techniques provide a precise and targeted approach to modify gene(s) responsible for milk allergenicity without compromising milk qualities^5^.

In farm animals, the combinations of GE and assisted reproductive technologies (ARTs) have been used for the production of targeted gene-edited animals. Among the livestock, cattle and buffaloes have long generation intervals and are monotocous, i.e., yield one offspring per calving. In these species, somatic cell nuclear transfer (SCNT) is the preferred ART for producing GE animals. Over the last decade, SCNT has been efficiently used to produce several gene-edited farm animals^6^. In buffalo, our group produced more than 20 cloned buffaloes using optimized SCNT method^7^. However, very limited attempts have been made to produce gene-edited cloned embryos in buffaloes and no live calf was produced yet. Recently, our group reported the successful production of *MSTN*-gene edited cloned embryos of buffalo^8^.

For modifying milk compositions, previous studies highligthed the production of *BLG* edited cells, embryos, and animals (cows and goats) using GE technologies^9–12^. However, before this study, no attempt was made in buffalo to edit *BLG*. To address this gap, we utilized the CRISPR/Cas system and SCNT method to generate *BLG*-edited single cell clones and their use to produce cloned embryos. This study provides evidence of the utility GE in buffaloes and offer promising prospects for producing *BLG* knockout buffaloes in the future.

## Results

### Determination of the *BLG* editing efficiency in buffalo fibroblasts

**Figure 1:**
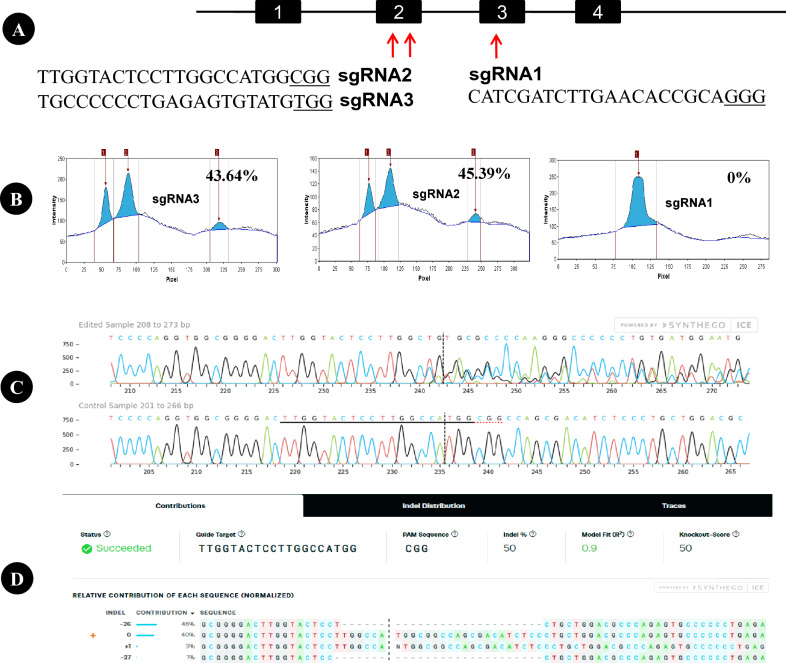
Buffalo *BLG* editing utilizing the CRISPR/Cas system. (**A**) Schematic representation of single guide RNAs (sgRNAs) targeting the second and third exons of buffalo *BLG*. The sgRNA sequences are depicted, with protospacer adjacent motif (PAM) sequences underlined. (**B**) Analysis of the T7E assay-based gel electrophoresis bands using the Gel Analyzer 19.1 software. Successful gene editing events indicate by three peaks at sgRNA2 and sgRNA3, while a single peak indicate no editing at sgRNA1. Gene editing percentage data are also shown. (**C**) Chromatogram nucleotide sequence data of cell pool edited with sgRNA2 (upper) and wild type cells (lower). Multiple overlapping peaks after the cutting site (vertical dashes line) of sgRNA2 in the edited cell pool and clear peaks in wild type cells. (**D**) Possible edited genotypes predicted by indels analysis (ICE, Synthego) along with the percentage of gene editing and knockout score.

We designed three sgRNAs to target the buffalo *BLG* (Fig. 1A), and assessed their editing efficiency in fibroblast cells. The T7E assay results have additional bands with expected sizes indicated successful editing of *BLG*. sgRNA2 exhibited 45.39% and sgRNA3 exhibited 43.64% editing rates (Fig. 1B). Conversely, sgRNA1 exhibited negative T7E assay results, which indicated its inefficacy to induce editing events at the *BLG* target site (Fig. 1B and supplementary Fig. 2). Further scrutiny of the transfected cells with sgRNA2 was conducted through Sanger sequencing and subsequent the Tracking of Indels by Decomposition (TIDE) and the Inference of CRISPR Edits (ICE) analyses (Fig. 1C and D). TIDE analysis identified 51.8% editing efficiency, and ICE analysis identified 50% editing efficiency. Based on these analyses, sgRNA2 was selected for subsequent experiments.

### Establishment of single cell clones of *BLG*-edited fibroblasts and their analysis

**Figure 2:**
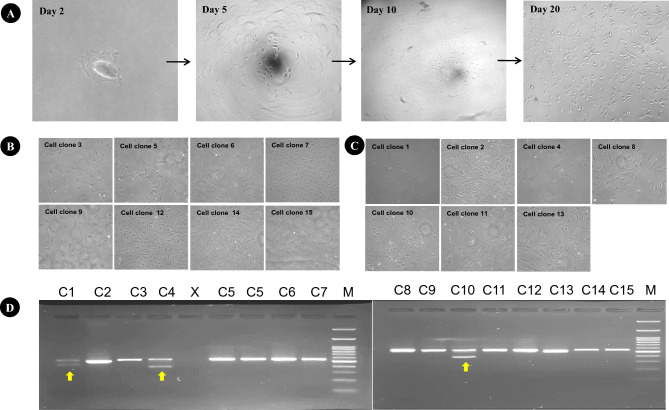
Establishment of single cell clonal lines and their screening using PCR gel electrophoresis. (**A**) In-vitro growth of a single cell recorded at different days in culture. (**B**) 8 wild type single cell clones and (**C**) 7 *BLG*-edited single cell clones. It can be noticed that some wild type and *BLG*-edited clones have vacuolation and abnormal morphology, particularly C5 and C10. (**D**) PCR-based screening of established single cell clones. Gel electrophoresis of the PCR products revealed that three colonies, denoted as C1, C4, and C10, exhibited two bands, indicative of large deletions at the *BLG* target site. C represent cell clone(s), X represents no sample, and M represents 100 bp ladder.

**Figure 3:**
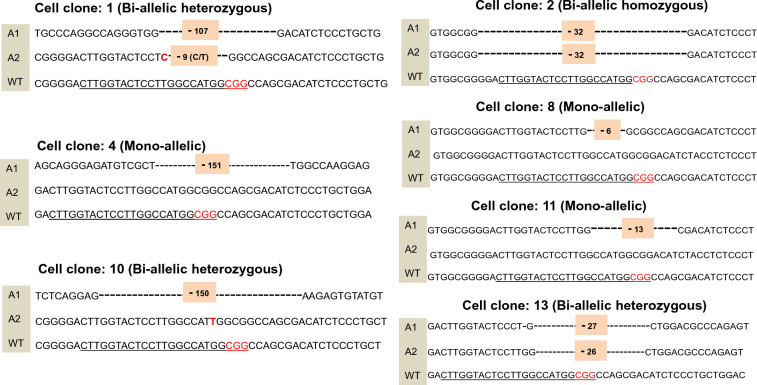
Gene editing detection in single cell clones using by TA cloning and Sanger sequencing. Cell clone 1 has the bi-allelic heterozygous mutation (−/−), one allele with − 107 bp deletion and the other allele with − 9 bp deletion with C/T conversion; cell clone 2 has bi-allelic homozygous mutation (−/−), both alleles were − 32 bp deletion, cell clone 4 has mono-allelic mutation (−/+), in which one allele with − 151 bp deletion, cell clone 8 has mono-allelic mutation (−/ +), in which one allele with − 6 bp deletion; cell clone 10 has bi-allelic heterozygous mutation (−/−), one allele with − 150 bp deletion and the other allele with one bp T insertion; cell clone 11 has mono-allelic mutation (−/+), in which one allele with − 14 bp deletion; and cell clone 13 has bi-allelic heterozygous mutation (−/−), one allele with − 27 bp deletion and the other allele with − 26 bp deletion.

The generation of single cell clones is a crucial step in the production of CRISPR-based edited cloned embryos and animals by SCNT method. In this study, we established 15 single cell clones, and noticed that some of the colonies have high level of vacuolation and abnormal morphology (Fig. 2A–C). To confirm the *BLG* editing in these established clones, PCR-based screening was performed. Gel electrophoresis of the PCR products revealed that three colonies, denoted as C1, C4, and C10, exhibited two bands, indicative of large deletions at the target site (Fig. 2D). Subsequent Sanger sequencing confirmed editing events in seven single-cell clones (C1, C2, C4, C8, C10, C11, and C13). To detect allele specific information, we employed TA cloning and Sanger sequencing analysis of these seven single cell clones. These seven clones exhibited various types of editing, including bi-allelic (−/−) heterozygous, bi-allelic (−/−) homozygous and mono-allelic (−/+) gene editing, each involving distinct nucleotide deletions and insertions (Fig. 3). Off-target (OT) effects are a significant concern in CRISPR/Cas9 applications. We screened five potential OT sites across three single-cell clones (C2, C10, and C13). Sanger sequencing revealed no detectable OT effects in our *BLG*-edited cell clones (Supplementary Fig. 2).

### Bioinformatic prediction of *BLG*-edited mutations

**Figure 4:**
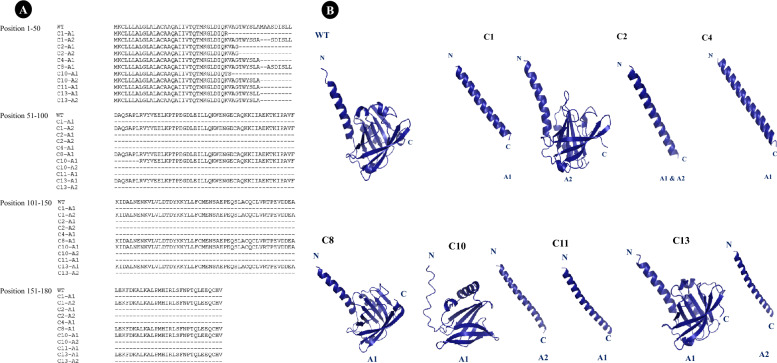
Prediction of protein sequences and 3D structures of BLG in single cell clones. (**A**) The multiple alignments of predicted BLG amino acid sequences, and their comparison with wild type BLG. The fate of *BLG* edited sequences as follows, C1-A1: early truncation, resulting in a 32 amino acid peptide and C1-A2: minor deletion, maintaining a structure similar to the wild type (177 aa); C2: bi-allelic homozygous truncation (−/−), yielding a 35 amino acid peptide; C4: mono-allelic (−/+) edited clone with early truncation, forming a 41 amino acid peptide; C8: mono-allelic (−/+) edited clone with a 2 amino acid deletion, producing a 178 aa protein; C10-A1: 24 amino acid deletion, retaining a distinct structure from the wild type and C10-A2: early truncation, resulting in a 41 amino acid peptide; C11: mono-allelic truncation (−/+), generating a 41 amino acid peptide; C13-A1: 9 amino acid deletion, yielding a 171 amino acid protein and C13-A2: early truncation, producing a 41 amino acid peptide. (**B**) 3D-structure perdition using AlphaFold2. It can be noticed that C1A1, C2A1, C2A2, C4A1, C10A2, C11A1, and C13A2 clones have early truncation coding frame, and, C1A2, C8A1, C10A1, and C13A1 has slightly disoriented proteins. (**C**) Represents carboxy terminal, N represents amino terminal. A1 and A2 represent allele first and second of *BLG*, respectively. WT represents wild type (+/+).

We also aimed to analyze, validate, translate, and predict the 3D structure of edited nucleotide sequences of *BLG*. ExPASy translated the BLG amino acid sequences, and their ORFs aligned with the wild-type BLG protein sequence to identify fate of gene editing (Fig. 4A). AlphaFold2 was employed for 3D protein structure prediction, considering insertion, deletion, and substitution events. AlphaFold2 predicted distorted structures for sequences with non multiple of 3 deletion or insertion events (C1A1, C2A1, C2A2, C4A1, C10A2, C11A1, and C13A2), causing coding frame shifts and truncation. In contrast, sequences with multiple of 3 events (C1A2, C8A1, C10A1, and C13A1) resulted in complete but slightly disoriented proteins due to amino acid deletions (Fig. 4B).

### Generation of cloned embryos from *BLG*-edited fibroblasts

**Figure 5:**
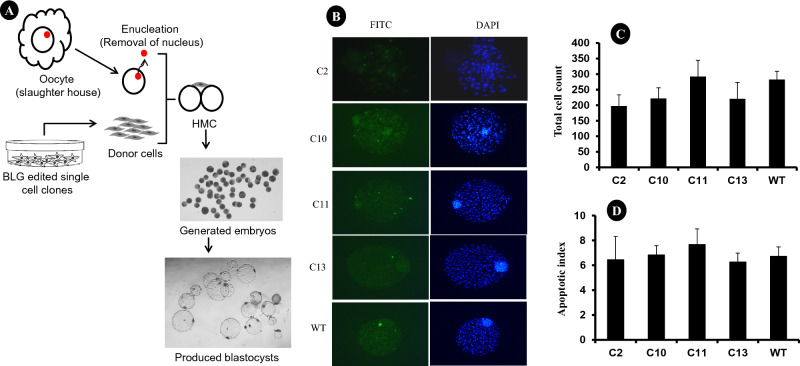
Production of *BLG*-edited cloned embryos and assessment of their quality. (**A**) Schematic representation of production of cloned embryos using HMC method of SCNT. (**B**) Determination of total cell count and apoptosis index of produced cloned blastocysts using TUNEL assay. FITC conjugated dUTP-labelled nuclei considered as apoptotic, whereas DAPI-stained nuclei used for determining total cell count. The total cell count (**C**) and apoptotic index (**D**) of blastocysts produced using *BLG*-edited cells and wild type cells. HMC represents handmade cloning. (**C**) In bar chart, represents *BLG*-edited cell clones and WT represents wild type cells.

We used 4 different *BLG*-edited cell clones as donor cells for the generation of cloned embryos through handmade cloning (HMC) method of SCNT (Fig. 5A). In each HMC experiment involved the utilization of two different *BLG*-edited cell clones alongside wild-type fibroblasts. The in vitro developmental competences of embryos were recorded at day 8 (Table 1). To assess the health of the produced blastocysts, we performed the TUNEL assay (Fig. 5B). The results indicated that there were no significant differences in terms of total cell count and apoptotic index between edited donor cells-derived cloned blastocysts and wild-type cloned blastocysts (Fig. 5C and D).

**Table 1:** In-vitro developmental competence of cloned embryos produced using *BLG*-edited donor cells.

Cell type	Editing status	SCNT embryos, n	Blastocyst rate (%)
Wild type	No-editing (+/+)	105	46 (43.80%)
C-2 colony	Bi-allelic editing (−/−)	150	62 (41.33%)
C-13 colony	Bi-allelic editing (−/−)	132	51 (38.63%)
C-10 colony	Bi-allelic editing (−/−)	90	26 (28.88%)
C-11 colony	Mono-allelic editing (+/−)	115	49 (42.60%)

## Discussion

Milk is a prominent nutritional source for humans, yet certain individuals, particularly infants, may experience allergies to milk and its derivatives^3^. Notably, the BLG, a protein found in ruminant milk, has been identified as an allergen, however, this protein is absent in human milk^15^. With advancements in GE technologies, several researchers have attempted to edit the *BLG* that aims to eliminate this allergenic component from ruminant milk^9–12,16–18^. BLG-free cow milk exhibited diminished allergenicity in Balb/c mice, with reduced allergic responses such as rectal temperature drop and allergen-specific IgE production^11^. Also, IgE binding from cow milk allergy patients to BLG-free milk significantly decreased in comparison to control milk. Thus, GE approach holds promise for addressing milk allergies and enhancing the overall safety and tolerability of dairy products for individuals having BLG sensitivities. Based on these findings and importance of buffalo milk globally, we edited *BLG* to produce embryos via SCNT. This study offers a pathway for generating buffaloes yielding BLG-free milk.

Three sgRNAs were designed for CRISPR-based editing of the BLG gene, with sgRNA2 showing favorable outcomes, as demonstrated  by T7E and Sanger sequencing results. Considering the variability in sgRNA efficacy, it's prudent to assess multiple sgRNAs for target genes^19^. Electroporation was chosen as the transfection method due to its speed, simplicity, and scalability^20^. In this study, the overall editing rate was 46.7%, which aligns with rates in farm animals. For instance, Bajwa et al.^21^ achieved 33% editing rates, Dua et al.^8^ achieved 25% editing rates, and Su et al.^22^ achieved 72% editing rates in buffalo fibroblasts, Wang et al.^23^ achieved 21% editing rates in pig fibroblasts, and Ishino et al.^24^ achieved 11.3% editing rates in cattle fibroblasts. The efficiency of gene editing is control by several factors such as GC contents of PAM, nucleotides sequence at the sgRNA spacer, secondary structure of sgRNA, chromatin state, and transcriptional stage of targeted gene^25^. Therefore, understanding and engineering the CRISPR/Cas9 system is crucial. Despite advancements, achieving maximum genome-editing efficiency with minimal off-target effects remains challenging. To assess potential off-target mutations, we Sanger sequenced five potential off-target sites in three cell clones (C2, C10, and C13). The sequencing results revealed absence of off-target modifications in all three clones. Thus, this study yields precise *BLG*-edited single-cell clones for SCNT applications in buffalo.

Establishing single-cell clonal populations is challenging and tedious task. Common methods like cloning rings and limiting dilution have drawbacks such as mixing of cells^26^. We opted for the manual pick-up method, in which manually transferring single cells into each well of a 96-well plate under microscopic control. This approach was previously used to establish single cell clonal population from electroporated buffalo cells^8,21^. We established 15 single-cell clones, of which 4 clones displaying bi-allelic (−/−) and 3 clones displaying mono-allelic (−/+) editing. We also observed that extended culture condition led to vacuolation and abnormal morphology in both edited and non-edited clones^24^. Addressing this, we propose supplementing the culture medium with growth factors like ITS, EGF, and FGF to stimulate fibroblast proliferation.

We conducted a comprehensive analysis of nucleotide sequences of seven *BLG*-edited cell clones. Our findings revealed distinct structural outcomes for edited sequences that influenced by the nature of deletion or insertion events. This aligns with the frame preserving nature of multiples of three insertions or deletions that allow the maintenance of the correct reading frame during translation^27^. The similar predictions have been made by other researchers^28^. Based on these prediction data, bi-allelic cell clone C2 can be most preferred for the production of live *BLG*-edited buffaloes through SCNT. As cryopreservation allows the long term storage of cells, we cryopreserved all these cell clones for future applications.

Application of GE for buffalo embryo production presents challenges, especially the choice between embryo production methods such as zygote microinjection and SCNT. Zygote microinjection often yields mosaic embryos and low gene editing rates, thus, posing difficulties in generating homozygous animals in long gestation period animals, such as buffalo^29–32^. In contrast, SCNT employs pre-screened gene edited cells as donors, holds promise for producing GE animals with desired genetic traits without the issue of mosaicism^31,34,35^. Our previous work utilized the HMC method for SCNT as it eliminates the need for expensive micromanipulation equipment and skilled personnel, making it an attractive option for GE applications in buffalo^8^. In this study, blastocyst rates were comparable among wild type embryos (43.80%) and edited clones such as bi-allelic (C-2) at 41.33  and (C-13) at 38.63%, and mono-allelic (C-11) at 42.60%. These findings imply that *BLG* editing does not significantly affect early embryonic development, and the results are agreeing with observations in other farm animal species^9,11,18^. However, mono-allelic clone C-10 exhibited lower blastocyst production (28.88%). The low blastocyst production rates of C10 could be due to a high level of vacuolation and abnormal cell morphology. It has been reported that vacuolation level and abnormal morphology of donor cells significantly affected the production of blastocysts^8,33,36^. The quality of embryos plays a pivotal role in determining the success of pregnancy establishment of in-vitro produced embryos^37,38^. The TUNEL assay was performed to assess the health of *BLG*-edited cloned embryos. There were no significant differences in total cell count and apoptotic index between *BLG*-edited and wild type cloned blastocysts. The TUNEL assay results indicated that there are no significant differences in total cell count and apoptotic index between *BLG*-edited and wild-type cloned blastocysts. However, further experiments are needed to determine the *in-vivo* developmental competence of these edited embryos and their suitability for live gene-edited buffalo production.

## Conclusion

We demonstrated the successful bi-allelic (−/−) editing of the *BLG* gene in buffalo cells through CRISPR and production of *BLG*-edited blastocyst stage embryos by SCNT. To our knowledge, this is the first report that demonstrated the production of *BLG*-edited embryos in buffalo. With the CRISPR system described herein, our long-term goal is to produce gene-edited buffaloes with BLG free milk traits to address milk allergy concerns.

## Methods

### Ethics statements

All experiments were performed according to the ethical standards of the institute. Animal procedures including biopsy collection were approved by the Institute Animal Ethics Committee, ICAR-National Dairy Research Institute, Karnal, India. The study is reported in accordance with ARRIVE guidelines, and approved by Institutional Biosafety Committee (IBSC).

### CRISPR single guide RNA (sgRNA) design and transfection of buffalo fibroblasts

To edit *BLG* in buffalo, we used CHOPCHOP software (https://chopchop.cbu.uib.no/) with *BLG* gene ID, default NGG PAM, and 20 nucleotide sgRNA length settings to design three sgRNAs targeting exon 2 and 3 of the *BLG*. The designed sgRNA candidates were *in-vitro* transcribed using precision gRNA synthesis kit (Thermo Scientific#A29377) as per the manufacturer’s instructions, and then co-transfected with Cas9 protein (Thermo Scientific#A36498) into newborn female buffalo fibroblasts that were established as per our lab protocol^13^. The cells at passage 2 were transfected using the Amaxa nucleofector reagents (Lonza, Basel, Switzerland), according to program EN150 as per the manufacturer’s guidelines. Transfected cells were cultured for 72 h, and then, collected for genomic DNA extraction and generation of single cell clonal lines. The T7E1 assay and Sanger sequencing were performed to verify status of targeted *BLG* gene editing.

### Detection of editing rates

The genomic DNA from transfected cells was extracted utilizing the Wizard genomic DNA purification kit, (Promega#A1125) as per the manufacturer’s instructions. The *BLG* primers (for exon 2, F- TGCCCCTCAAATTTTCCCCA and R-AAAGCCCTGGATAAGCAGCC; and for exon 3, F-CTGGCCCTCAGTTCATCCT and R-AGCAAAGAGAGCTCGGGTGT) were designed using PRIMER3 software (http://bioinfo.ut.ee/primer3-0.4.0/) and homology checked through nblast against nucleotide database of NCBI. The target sequence was subsequently amplified through polymerase chain reaction (PCR) under the following conditions: initial denaturation at 94 °C for 5 min, followed by 40 cycles at 94 °C for 20 s, 60 °C for 30 s, 72 °C for 35 s, and a final extension at 72 °C for 5 min. The PCR products from each sample underwent assessment using the T7E1 assay to detect editing in the *BLG* gene. Following denaturation and annealing of PCR products in NEB buffer 2 using a thermocycler, the hybridized PCR products underwent digestion with T7 endonuclease 1 (NEB#M0302L) for a duration of 20 min at 37 °C. The digested products were then subjected to 2% agarose gel electrophoresis. The gel images were analysed using the Gel Analyzer 19.1 software (www.gelanalyzer.com). The editing rate was determined by employing the formula: efficiency = [(sum of cleaved band intensities/(sum of cleaved and parental band intensities)] × 100. For TIDE and ICE analysis, PCR products were submitted for Sanger sequencing and resulted files were uploaded to the TIDE web tool (http://tide.nki.nl) and the ICE web tool (https://ice.synthego.com) for analysis. Both algorithms provided the percentage of insertions and deletions (indels).

### Establishment of single cell clones and their genotype analysis

After 72 h of transfection, cells were trypsinized and the cell pellet was suspended in culture medium. The resuspended cells were then dispersed at low density in 3 mL of culture medium in a 35-mm culture dish. Single cells were manually picked using a fine-pulled glass capillary under optical control with a stereo zoom microscope and transferred into individual wells of a 96-well plate (one cell per well). The culture medium was supplemented with 10 ng/mL epidermal growth factor (Gibco#PHG0311) to enhance proliferation, and the EGF-supplemented culture media were refreshed every fifth day. Regular examination of cell clones was conducted using an inverted microscope, and morphology images were captured. Subsequently, cells from each clone were expanded, analyzed, and cryopreserved for future use. In the established single cell clones, *BLG* editing was assessed by analyzing targeted *BLG*-specific PCR products. These PCR products from each single cell clone were ligated into the pJet1.2/blunt TA cloning vector using the Clone JET PCR cloning kit (Thermo Scientific#K1232). Following this, the cloned PCR products were transformed into *E.coli* cells, and plasmids were isolated from 8 to 10 *E. coli* colonies using a geneJET plasmid miniprep kit (Thermo Scientific#K0503). The isolated plasmids underwent the Sanger sequencing, and the obtained data were analyzed to determine the actual editing genotypes.

### Off-target analysis

We computationally predicted potential off-targets (OTs) of sgRNA2 using Cas-Offinder (http://www.rgenome.net/cas-offinder/), aligning with the buffalo genome assembly (NDDB_SH_1). Five OTs containing NGG PAM were selected (supplementary table 1 and 2). Off-target sites were amplified via conventional PCR, followed by Sanger sequencing. Analysis were done on three single cell clones, namely C2, C10, and C13, to detect any potential off-target effects.

### Protein structure prediction from edited sequences

Firstly, we retrieved the nucleotide sequence (Accession no. AJ005429.1) and the corresponding amino acid sequence (Accession no. CAA06532.1) of the wild type *BLG* from the NCBI database. We targeted exon 2 of the *BLG*, and edited DNA sequences were placed into the corresponding wild type nucleotide sequences to generate the complete coding sequences. Subsequently, we utilized the ExPASy translation tool (https://web.expasy.org/translate/) to predict the open reading frames (ORFs) of the edited nucleotide sequences. To identify suitable ORF templates for further analysis, we performed the BLASTp, and compared the edited ORFs and the wild-type BLG protein (P02755, available at the UniProt database). This step aimed to select the appropriate templates for 3D structure prediction. Multiple alignments of the predicted amino acid sequences and the wild-type BLG were carried out using T-coffee (https://www.ebi.ac.uk/Tools/msa/tcoffee/). The protein structure prediction relies on the alignment between the wild type *BLG* sequence and edited ORF templates. For the actual 3D structure of the predicted amino acid sequences, we employed AlphaFold2, a highly efficient deep learning and artificial intelligence based tool (https://alphafold.ebi.ac.uk/). The resulting predicted structures were visualized using the PyMOL molecular graphics system, Version 2.0, by Schrӧdinger LLC (https://pymol.org/2/).

### Production of cloned embryos

The handmade cloning (HMC) method, which was reported by us^14^, was employed for the production of *BLG*-edited cloned embryos. Briefly, buffalo ovaries were collected from a local abattoir and transported to the laboratory in normal saline within 4–6 h, and oocytes were harvested. In-vitro matured oocytes were denuded and subjected to zona pellucida removal, followed by manual bisection of protrusion cone-bearing oocytes. For nuclear transfer, donor cells, comprising *BLG*-edited fibroblasts, were paired with enucleated cytoplasts using phytohemagglutinin. The wild type fibroblasts were used as control. The resulting couplets underwent a single step electrofusion to form the triplets composed of a demi-oocyte, somatic cell, and another demi-oocyte. After 4 h incubation, the reconstructed oocytes were activated with calcimycin A23187, followed by 6-dimethylaminopurine treatment. The activated embryos were cultured in Research Vitro Cleave medium (K-RVCL-50, Cook®, Australia) supplemented with 1% fatty acid-free BSA in 4-well dishes, with 15–20 embryos per well. The dishes were covered with mineral oil and kept undisturbed in a CO_2_ incubator for 8 days. On day 8, blastocyst production rates were evaluated as indicators of in-vitro developmental competence.

### Evaluation of embryo quality

For the evaluation of embryo quality, day 8 blastocysts were assessed for the total cell count and apoptosis index through TUNEL staining. Blastocysts were washed thrice with Dulbecco's phosphate-buffered saline (DPBS) containing 0.3% polyvinyl alcohol (PVA) in a 4-well dish and fixed in 4% paraformaldehyde for 1 h at 37 °C. After additional washes, the blastocysts were stored at 4 °C until staining. Permeabilization was achieved by incubating blastocysts with 0.5% triton X-100 for 40 min, followed by incubation with FITC-conjugated dUTP and terminal deoxynucleotidyltransferase (TdT) for 90 min at 37 °C in the dark. Subsequently, the embryos were treated with nuclear staining solution (10 µg/mL Hoechst 33342 and 50 µg/mL RNase in DPBS + 0.3% PVA) for 25 min at 37 °C. Stained blastocysts were mounted on glass slides, and images were captured using both UV and green filters to examine nuclei and apoptosis sites, respectively. Digital images obtained from an inverted fluorescence microscope were used for analysis. The apoptotic index for each blastocyst was calculated as follows: Apoptotic index of blastocyst = (number of TUNEL-positive nuclei/total number of nuclei in blastocyst) × 100.

### Statistical analysis

Statistical analysis was conducted using GraphPad Prism 5 software. The datasets underwent analysis through one-way analysis of variance (ANOVA), followed by the Tukey test for post hoc comparisons. Percentage values were subjected to arcsine transformation before analysis. Statistical significance was considered at a threshold of P < 0.05. The results are presented as mean ± standard error of the mean (SEM).

## Supplementary Information

Supplementary Figure 1.

Supplementary Figure 2.

Supplementary Tables.

Supplementary Legends.

## Data Availability

The sequencing dataset of *BLG* target region generated during the current study is available in the NCBI-Gen Bank repository (Accession numbers: OQ851625.1).
