# Author Correction: CRISPR-mediated editing of β-lactoglobulin (*BLG*) gene in buffalo

**DOI:** 10.1038/s41598-024-72295-1

**Published:** 2024-09-10

**Authors:** Aseem Tara, Priyanka Singh, Devika Gautam, Gaurav Tripathi, Chirag Uppal, Shreya Malhotra, Sacchinandan De, Manoj K. Singh, Bhanu P. Telugu, Naresh L. Selokar

**Affiliations:** 1 Animal Biotechnology Division (ABTD), ICAR-National Dairy Research Institute, Karnal, Haryana 132001 India; 2 Division of Animal Science, University of Missouri, Columbia, MO 65211 USA

Correction to: *Scientific Reports* 10.1038/s41598-024-65359-9, published online 27 June 2024

The original version of this Article contained an error in the Acknowledgements section.

“This work was supported by a grant, BMGF/802/1014522, from the Bill & Melinda Gates Foundation, USA, and partially by a grant, NASF/GTR-8025, from the Indian Council of Agricultural Research, New Delhi, India.”

now reads:

“This work was supported by a grant from the Bill & Melinda Gates Foundation, USA (Grant No. BMGF/INV-044234), and partially by a grant from the Indian Council of Agricultural Research, New Delhi, India (Grant No. NASF/GTR-8025).”

The original Article has been corrected.

