# Supplementary figures and images for: CRISPR-mediated editing of β-lactoglobulin (BLG) gene in buffalo
Source: Sci Rep. 2024 Jun 27;14:14822. doi: 10.1038/s41598-024-65359-9 (PMC11211398; doi:10.1038/s41598-024-65359-9)

## Slide 1
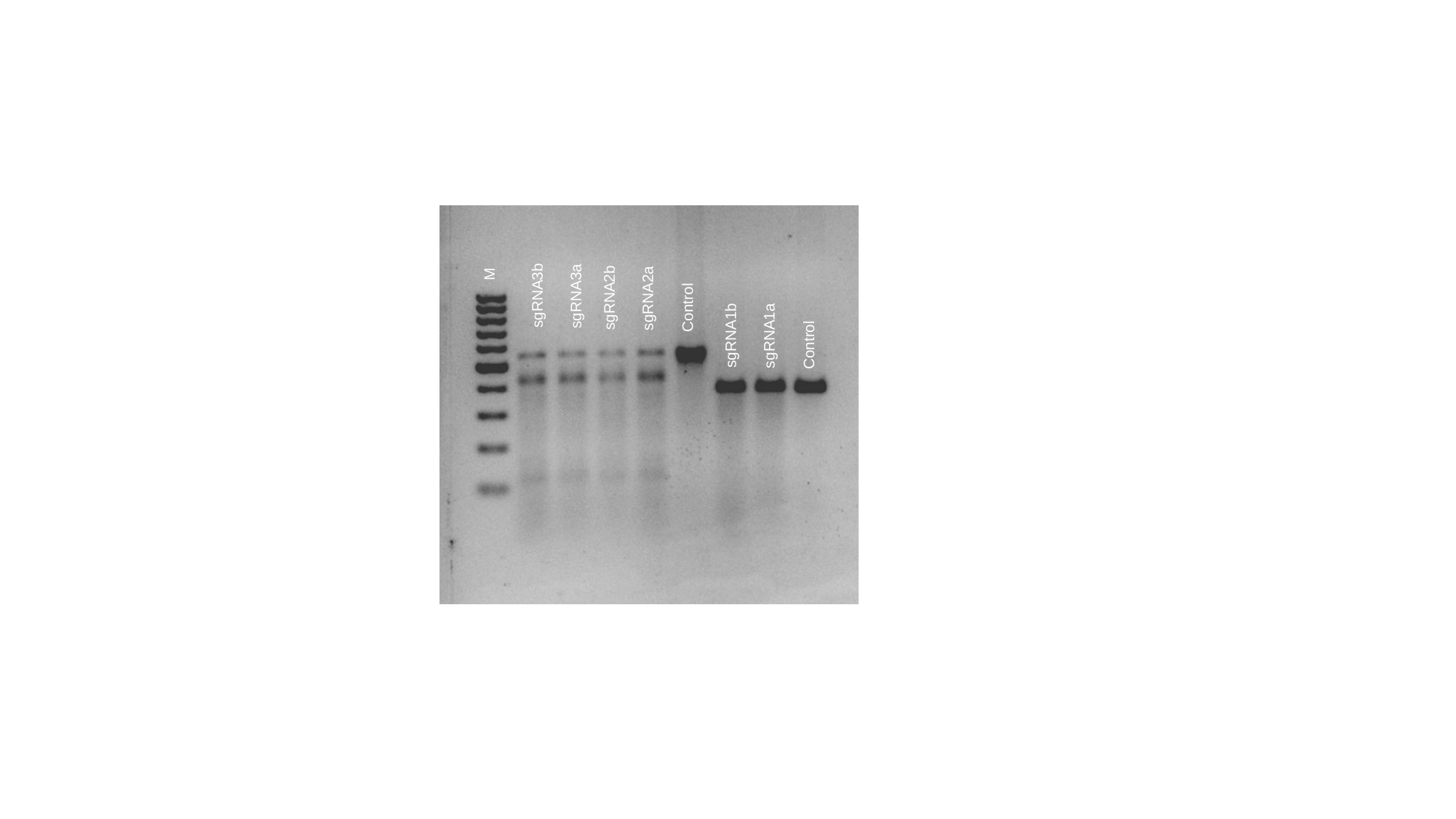

M
sgRNA3b
sgRNA3a
sgRNA2b
sgRNA2a
Control
sgRNA1b
sgRNA1a
Control

Supplement: Supplementary file 1 — Supplementary Figure 1. [file 41598_2024_65359_MOESM1_ESM.pptx]
