# Supplementary material for: CRISPR-mediated editing of β-lactoglobulin (BLG) gene in buffalo
Source: Sci Rep. 2024 Jun 27;14:14822. doi: 10.1038/s41598-024-65359-9 (PMC11211398; doi:10.1038/s41598-024-65359-9)
Supplement: Supplementary file 2 — Supplementary Figure 2. [file 41598_2024_65359_MOESM2_ESM.pptx]

## Slide 1
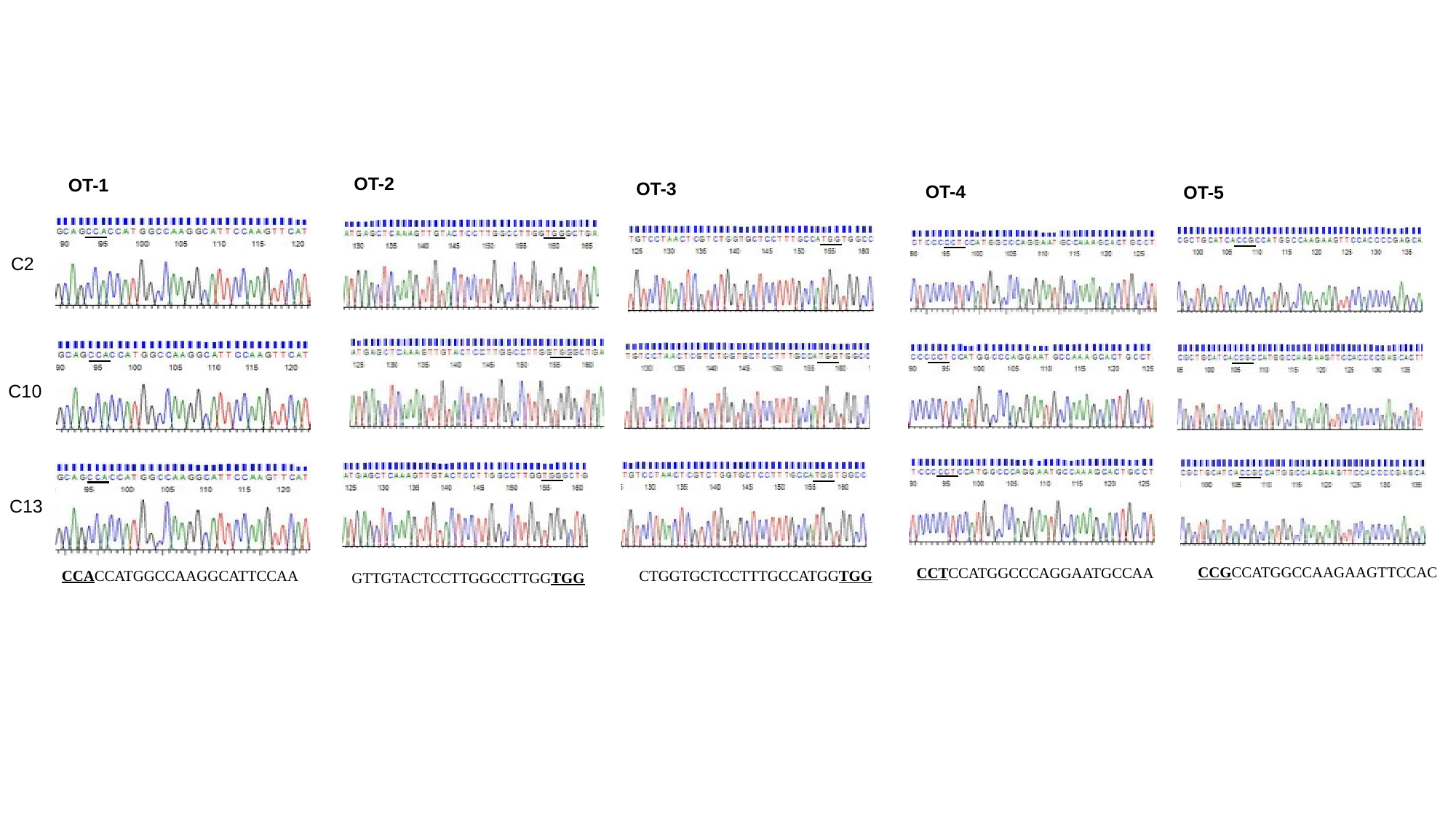

OT-2
OT-1
OT-3
OT-4
OT-5
C2
C10
C13
CCGCCATGGCCAAGAAGTTCCAC
CCTCCATGGCCCAGGAATGCCAA
CCACCATGGCCAAGGCATTCCAA
CTGGTGCTCCTTTGCCATGGTGG
GTTGTACTCCTTGGCCTTGGTGG
