# Supplementary material for: CRISPR-mediated editing of β-lactoglobulin (BLG) gene in buffalo
Source: Sci Rep. 2024 Jun 27;14:14822. doi: 10.1038/s41598-024-65359-9 (PMC11211398; doi:10.1038/s41598-024-65359-9)
Supplement: Supplementary file 3 — Supplementary Tables. [file 41598_2024_65359_MOESM3_ESM.docx]

| **Off-target** | **Off-target nucleotide site with PAM** | **Chromosomal location** | **Genomic position** | **Direction** |
| --- | --- | --- | --- | --- |
| OT-1 | TTGGAATGCCTTGGCCATGGTGG | CM034271.1 | 173489744 | - |
| OT-2 | GTTGTACTCCTTGGCCTTGGTGG | CM034275.1 | 30273830 | + |
| OT-3 | CTGGTGCTCCTTTGCCATGGTGG | CM034281.1 | 45120584 | + |
| OT-4 | TTGGCATTCCTGGGCCATGGAGG | CM034282.1 | 85251602 | - |
| OT-5 | GTGGAACTTCTTGGCCATGGCGG | CM034287.1 | 10115210 | - |

Table S1. Potential off-target sites of sgRNA2

| Off-target site |  | Nucleotide primer sequence | PCR product size |
| --- | --- | --- | --- |
| OT-1 primers | F | CCTGAAGAAAAGGCCAGGGA | 306 |
|  | R | GCTTTCTGTTGCCAGGAAAA |  |
| OT-2 primers | F | TGTGCACATCTCACACACCT | 335 |
|  | R | TTTCGCTTTGGCATCCATGG |  |
| OT-3 primers | F | CCACACTTATTCCCAGATGTCCT | 351 |
|  | R | CTGTGGTGTGATCAGGGCTT |  |
| OT-4 primers | F | ACCATCTGTACTGCAAGCTGA | 303 |
|  | R | GGAGGTGCCCTTTGGGTTT |  |
| OT-5 primers | F | CCAGCCTGAACAGCCCCC | 307 |
|  | R | CGCCTAGCAGAAGAGCTTGA |  |

Table S2. Primers used to amplify the off-target sites
