# Supplementary material for: CRISPR-mediated editing of β-lactoglobulin (BLG) gene in buffalo
Source: Sci Rep. 2024 Jun 27;14:14822. doi: 10.1038/s41598-024-65359-9 (PMC11211398; doi:10.1038/s41598-024-65359-9)
Supplement: Supplementary file 4 — Supplementary Legends. [file 41598_2024_65359_MOESM4_ESM.docx]

**Supplementary Fig. 1:** The original gel photo of the T7E assay was used to determine the editing success of the designed sgRNAs. The sgRNA samples were run in duplicate, labeled as sgRNA1a and sgRNA1b, and so on.

**Supplementary Fig 2:** The chromatogram displays potential off-target sites in three single-cell clones. The absence of overlapping peaks indicates no off-target editing, with sequences identical to the wildtype sequences. Off-target sites are highlighted with underlined PAM sequences for clear identification.
